# Pelvic Actinomycosis Mimicking Pelvic Malignancy

**DOI:** 10.1055/s-0039-1688462

**Published:** 2019-06-19

**Authors:** Sofia Modesto Saramago, Joana Catarina Cominho, Sara Soares Marques Proença, Pedro João Casado Conde, Filomena Maria Pinheiro Nunes

**Affiliations:** 1 Department of Obstetrics and Gynecology, Hospital de Cascais Dr. José de Almeida, Cascais, Portugal

**Keywords:** pelvic neoplasms, differential diagnosis, actinomycosis, intrauterine devices, anti-bacterial agents, neoplasia pélvica, diagnóstico diferencial, actinomicose, dispositivos intra-uterinos, antibióticos

## Abstract

Asymptomatic female genital tract colonization with *Actinomyces spp* is not uncommon, particularly among intrauterine device users. Pelvic actinomycosis is an extremely rare disease. The clinical picture can resemble an advanced ovarian malignancy. We report a case of pelvic actinomycosis mimicking ovarian malignancy diagnosed postoperatively. Preoperative diagnosis is possible if there is a high index of suspicion, obviating extensive surgery and preserving fertility, since long term antibiotic treatment can be completely effective. Pelvic actinomycosis should be included in the differential diagnosis of women presenting a pelvic mass, especially if there is intrauterine device use history.

## Resumo

A colonização assintomática do aparelho genital feminino por *Actinomyces spp* não é infrequente, sobretudo em utilizadoras de dispositivo intra-uterino. A actinomicose pélvica é uma doença extremamente rara. O quadro clínico pode assemelhar-se ao de uma neoplasia maligna do ovário avançada. Relatamos um caso de actinomicose pélvica, simulando uma neoplasia maligna do ovário, com diagnóstico pós-operatório. O diagnóstico pré-operatório é possível se houver um elevado grau de suspeição, permitindo evitar cirurgias extensas e preservar a fertilidade, uma vez que o tratamento antibiótico prolongado pode ser totalmente eficaz. A actinomicose pélvica deve ser incluída no diagnóstico diferencial da mulher que apresente uma massa pélvica, sobretudo se houver história de uso de dispositivo intra-uterino.

## Introduction

Pelvic actinomycosis is a rare, chronic, suppurative and granulomatous disease caused by the anaerobic Gram-positive bacteria *Actinomyces spp*, most commonly *Actinomyces israelii*. *Actinomyces* are commensal organisms that colonize the human oral cavity, gastrointestinal and genital tract.^1^ *Actinomyces* are normally unable to cross the mucosal barrier. Tissue injury, such as trauma, surgery, or foreign body, is required for progression from colonization to clinical infection. Once the mucosal barrier has been breached, the bacteria usually spreads by continuity (lymphatic and hematogenous spread are uncommon), invading surrounding tissues and originating abscesses and sinus tracts.

Abdominopelvic actinomycosis comprises ∼ 20% of reported cases of actinomycosis.^1^ Pelvic actinomycosis is predominantly associated with intrauterine device (IUD) use.^1^ The disease is characterized by a chronic, indolent course, typically presenting symptoms such as fatigue, fever, weight loss and lower abdominal pain, sometimes associated with a palpable mass.

The ability of this disease to mimic pelvic malignancy has been previously presented in several case reports and case series.^2^ ^3^ In most cases, the diagnosis of abdominopelvic actinomycosis is only established after exploratory laparotomy for suspected malignancy. It has been estimated that fewer than 10% of patients are diagnosed preoperatively.^1^

Medical treatment alone with penicillin is highly effective and can successfully eliminate pelvic actinomycosis, avoiding extensive extirpative surgery and preserving fertility.^1^

## Case Description

A 47-year-old woman was referred for gynecological evaluation by her attending gastroenterologist, after a pelvic mass was found on a computed tomography (CT) scan.

The patient was born in Brazil and had been living in Portugal for the past 26 years. Her medical history was unremarkable. She used a copper IUD for 6 years and she had it removed 4 months before. Her last pap smear, 2 years earlier, showed no abnormalities.

**Fig. 1:**
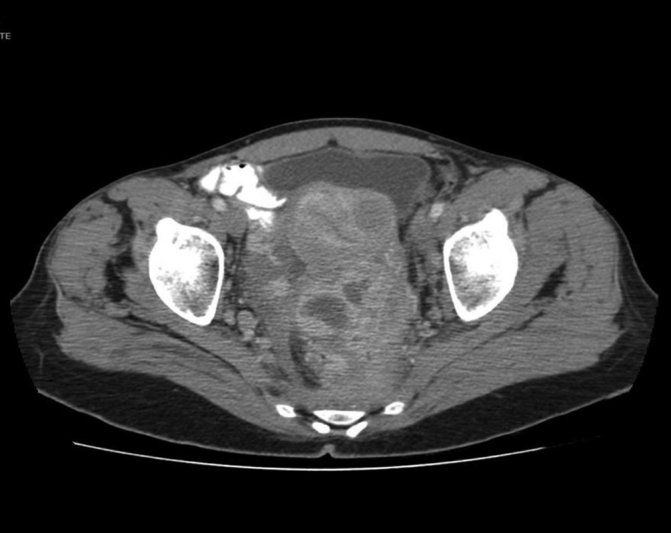
Axial abdominopelvic computed tomography scan image showing a large heterogeneous pelvic mass.

A concentric infiltrative rectal lesion was noted in a screening colonoscopy she had, given her family history of colorectal cancer. Biopsies of this lesion were inconclusive, without dysplasia. A CT of the thorax, abdomen and pelvis was ordered and revealed a large solid heterogeneous pelvic mass, left hydronephrosis and iliac lymph node enlargement (Fig. 1).

The patient had no complaints. At physical examination, a hard, tender, palpable mass in the posterior cul-de-sac was noted; no other abnormalities were detected.

A pap smear was obtained and the results were normal. Laboratory studies only revealed mild normocytic anemia (hemoglobin 10.8 g/dL, mean corpuscular volume 84.7 fL). The white blood cell count, erythrocyte sedimentation rate and C-reactive protein were normal; serum creatinine was normal (0.60 mg/dL). Tumor markers were also within the normal range (CA 125, CA 19.9, CEA).

**Fig. 2:**
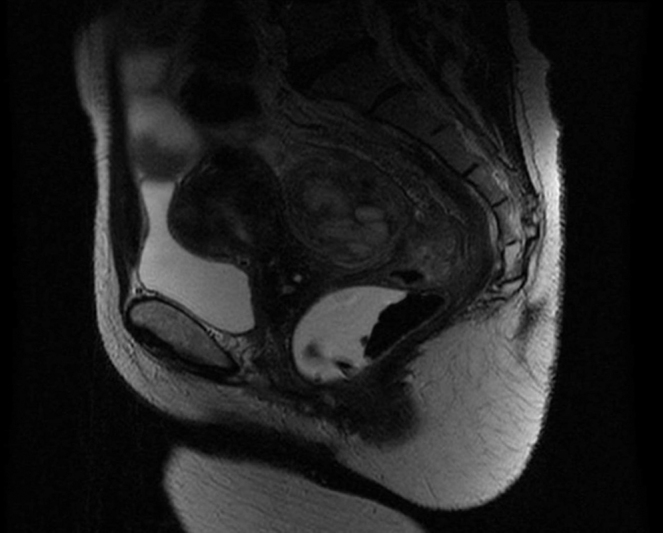
Sagital T2-weighted pelvic magnetic resonance image depicting the same large heterogeneous, mixed, retrouterine mass.

Pelvic magnetic resonance showed a heterogeneous, mixed, retrouterine mass, probably of left adnexal origin, measuring 6 × 5 × 5 cm (Fig. 2). The mass was predominantly solid, with cystic areas, and it seemed to have cleavage plane from the posterior uterine wall. High rectal and sigmoid colon concentric wall thickening was also apparent, and the mass involved the distal left ureter, resulting in left hydronephrosis. There was no ascites.

A malignant ovarian tumor was the leading diagnostic hypothesis. Alternative diagnoses considered were a primitive colorectal tumor with adnexal metastasis and deeply infiltrating endometriosis.

Definite diagnosis required histologic sampling of the mass. The patient had completed child-bearing and had no desire of preserving fertility. A multidisciplinary pelvic oncology team discussed the case. Given the ureteral and bowel compression, it was thought the mass required surgical resection, regardless of its etiology. A laparotomic approach was chosen, given the degree of suspicion of malignancy, the predominantly solid nature of the mass, its dimensions, and the level of expertise in laparoscopy at our center.

The surgical plan was discussed with the patient, who gave informed consent for left adnexectomy with intraoperative frozen section and a possible complete surgical staging procedure for ovarian cancer and even segmental bowel resection.

A left ureteric stent was placed prior to surgical intervention.

On exploratory laparotomy, a hard, left adnexal mass with ∼ 6 cm, extending to the rectum, sacrum and left pelvic wall was found. Given the infiltrative nature of the mass and the lack of cleavage planes with the nearby structures, and since the patient had no desire of preserving fertility, it was decided to complete total hysterectomy and bilateral salpingo-oophorectomy. Peritoneal lavage, total hysterectomy, right adnexectomy and *en bloc* resection of the described mass were performed. The resection was incomplete due to the lack of cleavage plane with the bony pelvis. The extemporaneous examination favored a benign inflammatory etiology and the surgery was concluded.

Final histopathological examination established a diagnosis of left tubo-ovarian actinomycosis, with active chronic inflammation and abscess formation.

Intravenous penicillin (5 million units every 6 hours) was administered for 4 weeks, followed by oral doxycycline for 12 months.

A follow-up CT of the abdomen and pelvis, 1 month after completing the penicillin course of treatment, showed no signs of the disease.

The patient had follow-up appointments about every 3 months for over 1 year, either with her attending gynecologic oncologist or infectiology specialist. During this time, she remained asymptomatic, except for troublesome vasomotor symptoms. Transdermal estrogen was prescribed with adequate relief.

## Discussion

Despite the association between IUD use and pelvic actinomycosis, asymptomatic genital tract colonization by *Actinomyces* in IUD users must be differentiated from clinically relevant pelvic actinomycotic infection.

*Actinomyces* exists in normal oral and gastrointestinal flora. Female genital tract colonization is also not uncommon. The incidence of *Actinomyces*-like organisms was 0.26% in a study of more than 20,000 pap smears.^4^ In this study, most women with positive *Actinomyces*-like organisms in cervical smears were IUD users (81%), with 60% having a copper IUD and 31% a levonorgestrel-releasing intrauterine system (LNG-IUS).^4^ *Actinomyces*-like organisms can be found in cervical smears in up to 7% of IUD users.^5^ ^6^ However, the pap smear lacks specificity in identifying *Actinomyces*, and only half the diagnosis made through pap smears are actually culture positive.^5^ ^7^

It is impossible to quantify the risk of developing serious pelvic infection in IUD users with *Actinomyces* genital colonization; however, it is probably exceedingly low.^5^ The finding of *Actinomyces* on a Pap smear is considered incidental and the asymptomatic patient does not require antimicrobial treatment or removal of the IUD.^5^ ^7^ ^8^

The rate of actinomyces-like organisms in cervical smears is lower with the more recent LNG-IUS than with the copper IUD.^4^ ^9^ The potential of LNG-IUS users with actinomyces-like organisms in cervical smears to develop pelvic actinomycosis is currently unknown. In a systematic review, including 83 cases of pelvic actinomycosis worldwide, between 1980 and 2014, 61 patients were IUD users. From these, 15 had a copper IUD, 2 had Lippes loop, and 2 had Dalkon Shield devices. Cases of pelvic actinomycosis with LNG-IUS use were not reported, but, in the majority of cases, the type of IUD used was not disclosed.^10^

In this case report, the patient had a copper IUD in place for 6 years, which had been removed 4 months before presentation. Although the development of pelvic actinomycotic abscesses in women with IUDs is exceptionally rare, the diagnosis should be considered in women of reproductive age with a pelvic mass, especially in those with an IUD in place or recently removed.^2^

Among women with pelvic actinomycotic abscesses, only 50% had a previous pap smear positive for *Actinomyces*-like organisms.^5^ In fact, our patient had no evidence of *Actinomyces* in two pap smears collected 2 years apart.

Given its rarity and lack of distinct clinical features, pelvic actinomycosis is a difficult diagnosis. Common symptoms include lower abdominal or pelvic pain, abnormal uterine bleeding or discharge, a palpable abdominopelvic mass, fatigue, weight loss, fever and symptoms related to bowel obstruction or obstructive uropathy. There may be anemia, mild leukocytosis, elevated C-reactive protein and elevated erythrocyte sedimentation rate.^1^ Imaging features are nondiagnostic and may be similar to those seen in other local inflammatory or neoplastic processes. Infiltration of adjacent tissues, across tissue planes, and sinus tract formation are characteristic of actinomycosis, although not specific.^1^

Several aspects make this case atypical in its presentation. The patient had no symptoms, and the pelvic actinomycotic abscess was incidentally discovered because a rectal infiltrative lesion was found during a screening colonoscopy. Also, iliac lymph node enlargement was reported on CT. Local or regional lymphadenopathy is unusual in actinomycosis.^1^ Mild normocytic anemia was the only relevant laboratorial finding in our patient and inflammatory markers were unchanged.

Pelvic actinomycosis clinically mimics ovarian cancer and other diseases, such as tuberculosis, pelvic inflammatory disease, lymphoma, inflammatory bowel disease, diverticulitis or endometriosis.

Correct nonsurgical diagnosis is possible by image guided biopsy or laparoscopic biopsy and prolonged antibiotic therapy can avoid extensive extirpative surgery and preserve fertility.^11^

A diagnosis of advanced ovarian cancer is often assumed and these patients are subjected to unnecessary exploratory laparotomy.^2^ ^3^ ^11^ The need of an intraoperative frozen section to distinguish actinomycosis and other benign processes from ovarian carcinoma must be emphasized to prevent complete staging procedures, pelvic and para-aortic lymphadenectomy, and the morbidity that follows.^2^

The final diagnosis is based on the recognition of actinomycotic sulfur granules on histology and/or on cultural identification of *Actinomyces*.^1^ Sulfur granules consist of clusters of actinomycetes and are highly suggestive, although not pathognomonic, of actinomycosis. Isolation through culture is more specific but challenging. *Actinomyces* are fastidious organisms, and less than 50% of suspected cases are confirmed by this method.^1^

High-dose prolonged therapy with penicillin has been the treatment of choice for actinomycosis. Traditionally, intravenous penicillin G at a dose of 18 to 24 million units a day is administered for 2 to 6 weeks, followed by oral penicillin V (2–4 g/day) for 6 to 12 months. Doxycycline, minocycline, clindamycin and erythromycin are considered reasonable alternatives for patients allergic to penicillin. Medical treatment can be completely effective.^1^ Since oral penicillin V is not available in our country, in this case, after 4 months of intravenous penicillin G therapy, a 12-month course of oral doxycycline was administered.

Surgical treatment alone is not curative but may be a useful adjunctive in selected cases. It also may be necessary if malignancy cannot be excluded.^1^

## Conclusion

Pelvic actinomycosis should be included in the differential diagnosis of pelvic masses suspicious for malignancy. Doctors should be especially aware of this disease when there is a history of IUD use. Non-surgical diagnosis of pelvic actinomycosis is possible, and antibiotic treatment can be completely effective, avoiding extensive surgery and preserving fertility. Pelvic actinomycosis is very rare; however, asymptomatic female genital colonization by this organism is not uncommon. The incidental finding of *Actinomyces*-like organisms on a pap smear does not require antimicrobial treatment or IUD removal.
